# Cytochrome nanowires under electron microscopy

**DOI:** 10.1042/ETLS20240008

**Published:** 2026-02-02

**Authors:** Holly A. Petersen, Allon I. Hochbaum, Daniel R. Bond, Fengbin Wang

**Affiliations:** 1 Department of Biochemistry and Molecular Genetics, University of Alabama at Birmingham, Birmingham, AL 35233, U.S.A.; 2 Department of Chemical and Biomolecular Engineering, Materials Science and Engineering, Chemistry, and Molecular Biology and Biochemistry, University of California Irvine, Irvine, CA 92697, U.S.A.; 3 Department of Plant and Microbial Biology, and BioTechnology Institute, University of Minnesota, Saint Paul, MN 55455, U.S.A.; 4 Gregory Fleming James Cystic Fibrosis Research Center, University of Alabama at Birmingham, Birmingham, AL 35233, U.S.A.

**Keywords:** cryo-EM, cytochrome nanowires, extracellular electron transfer, helical filament, microbial nanowires

## Abstract

Microbial nanowires are appendages that Bacteria and Archaea use to transfer electrons to external surfaces, such as minerals, electrodes, or other microbes. While initial studies suggested that nanowires were modified pili, recent advancements in cryo-EM revealed that microbial nanowires are composed of multi-heme *c*-type cytochromes. In this review, we discuss the discovery of microbial nanowires, advancements that allowed elucidation of their near-atomic resolution cryo-EM structures, and the impact of heme arrangement on electron transfer. We also discuss how new structural information can be used to identify filaments in images from published literature. The structural insights gained from these studies provide a deeper understanding of the mechanisms underlying long-range electron transport in microbial nanowires and their potential applications in bioelectronics and energy-generating microbial fuel cells.

## Introduction

In anoxic environments, many respiring bacteria can transfer electrons beyond their outer membranes to terminal acceptors, a process known as extracellular electron transfer [1]. While first discovered in the context of dissimilatory iron and manganese oxide reduction [2,3], this process also allows bacteria to utilize nearby syntrophic partner organisms such as methanogens, or even electrodes, for electron disposal [4–7]. In order to generate ATP via electron transport through the cytoplasmic membrane, mechanisms evolved to divert electrons out of the quinone pool to periplasmic carriers [8–12] and then across the insulating outer membrane to interact with extracellular electron acceptors [13–15]. At the exterior of the cell, redox proteins must then be able to make physical contact with a diverse range of minerals, electrodes, or partner organisms, unless electrons can be released onto soluble electron acceptors [16,17].

The hypothesis that conductive appendages solved the challenge of direct electron transfer beyond the cell surface was described first in *Geobacter sulfurreducens* [18]. Filaments sheared from the exterior of *Geobacter* cells demonstrated surprising conductivity [19–21], leading to these filamentous structures being termed ‘nanowires.’ Early *in silico* predictions suggested that nanowires could be constructed solely from a protein that resembled the N-terminal portion of the type IV pilin PilA (‘PilA-N’) [18]. This model was consistent with data showing that antibiotic insertional mutants did not display filaments or reduce Fe(III). However, a structure for this hypothetical ‘e-pili’ nanowire could not be obtained [22,23], prompting further investigation into the true identity of the observed nanowires.

Advancements in cryo-EM allowed detailed atomic structures to be determined for three different extracellular cytochrome filaments sheared from *G. sulfurreducens*. These 4–6 nm diameter nanowires share no sequence or structural homology, but all comprise linear, helical polymers of multi-heme c-type cytochromes [24–28]. However, the production of extracellular appendages and filaments is often dynamic, and different filaments might be secreted at the same time and are detectable under cryo-EM. For example, these cryo-EM studies find sheared samples from *G. sulfurreducens* commonly contain abundant 2–3 nm diameter DNA filaments and sometimes contains 7–8 nm type IV pili (T4P). This T4P is composed of equal parts PilA-N and the C-terminal portion of the pilin subunit (encoded downstream as PilA-C) to form a pilus that, while poorly conductive, may be essential for other roles such as extracellular sensing or secretion [29–31].

*G. sulfurreducens* has emerged as a model organism for extracellular electron transfer research, aided by the combination of available genome sequences and abundant genetic tools [32–35]. Due to the potential applications of cytochrome nanowires in fields such as bioelectronics and energy-generating microbial fuel cells, the structure and function of conductive nanowires are attracting significant scientific interest [33,36–38]. In this review, we will discuss the developments in cryo-EM that allowed high-resolution structural reconstruction of filaments from *Geobacter*, including the cryo-EM structures of multi-heme cytochrome nanowires OmcS, OmcE, and OmcZ; the impact of heme arrangement on electron transfer; and how specific image processing methodologies and tools have enabled further analysis of micrographs from earlier work.

### Evidence that *Geobacter* requires contact with insoluble electron acceptors

In the diverse sedimentary environments where *Geobacter* have been isolated, solid minerals like Fe(III) serve as a crucial terminal electron acceptor [39]. Early experiments with *G. metallireducens* and *G. sulfurreducens* showed that they could utilize both solubilized Fe(III) and insoluble Fe(III) oxides as terminal electron acceptors, with faster growth observed in the presence of soluble forms [3]. As a result, how do *Geobacter* cells reach these insoluble electron acceptors? Several observations indicated that electron transfer by *Geobacter* was occurring through direct contact with the insoluble electron acceptors, rather than via a mechanism involving a soluble chelator or redox shuttle. In experiments where physical contact between the bacteria and Fe(III) oxide was blocked, either by a dialysis membrane or by incorporation into microporous beads, Fe(III) reduction was not observed unless anthraquinone-2,6-disulfonate (AQDS) was added to the medium to act as an electron shuttle [3,40].

Additional evidence that *G. sulfurreducens* could build conductive linkages between cells came from biofilms grown on anode surfaces [21] and correlations between biofilm conductivity and current production [41]. As cells grew on electrodes, additional cell layers continued to contribute to current production, even when bacteria were over 20 microns from their electron-accepting electrode surface [42–44]. Studies with *G. sulfurreducens* biofilms on interdigitated electrodes observed conductivity at micron-scale distances as soon as cells could be observed spanning the two electrodes. Unlike metallic conductors that function over a broad voltage range, electron transfer in these biofilms peaked within a narrow window (approximately −300 to −100 mV vs. SHE). This behavior is characteristic of biological electron hopping, which requires a specific potential where redox cofactors are available to both accept and donate electrons. This match between the conductivity window and the known redox range of *Geobacter* cytochromes strongly suggested the involvement of a redox protein as the rate-limiting step in biofilm conductivity [45,46]. Additionally, medium exchange experiments confirmed that *Geobacter* biofilms do not rely on soluble redox mediators. In contrast with *Shewanella* biofilms, where removing the medium washes away essential flavin shuttles, replacing the medium of *Geobacter* biofilms had no effect on conductivity. This confirmed that the electron carriers in *Geobacter* are bound to the biofilm matrix rather than soluble [7,17].

### Identifying microbial appendages from near-atomic resolution cryo-EM maps

As evidence accumulated for extracellular conductivity in *Geobacter* strains, a lack of molecular data and nanowire structures led to multiple hypotheses and theoretical proposals.

The introduction of direct electron detectors in cryo-EM around 2012 instigated a ‘resolution revolution’ in the field of structural biology. As these detectors became more commonplace, the number of near-atomic resolution structures submitted to the protein data bank exploded [47–49]. User-friendly software packages, such as RELION [50] and cryoSPARC [51], further streamlined the process of three-dimensional (3D) reconstruction with improved resolution.

At its core, the cryo-EM workflow transforms thousands of low-signal, two-dimensional (2D) projections of a sample into a high-resolution, 3D map. Because biological molecules are susceptible to radiation damage, they are imaged in a thin layer of vitreous ice using a very low electron dose. This results in ‘noisy’ individual images where structural details are difficult to distinguish. To overcome this limitation, researchers extract thousands of individual filament segments and group them via reference-free 2D classification to separate different appendage types based on visual similarities. The subsequent 3D reconstruction is a highly iterative process. Because the relative orientations of the 2D projections are initially unknown, they must be determined by comparing the raw images against a low-resolution reference model. Each iteration of this ‘projection matching’ strives to improve the accuracy of these orientation assignments; as those assignments become more precise, the resulting 3D map reaches higher resolution. The process continues until the assignments no longer change significantly, at which point the reconstruction has converged to the final map. The general workflow, challenges, and opportunities of cryo-EM have been thoroughly discussed in various resources, including those reviews [52–56] and online courses such as Cryo-EM/ET 101 (https://cryoem101.org/).

When cryo-EM maps reach a resolution of ~4.1 Å or better, clear separation of β-sheet strands and unambiguous tracing of the protein backbone typically becomes feasible. Given that an organism’s genome encodes a finite and known set of protein sequences, it is possible to identify a protein *de novo* from the organism’s proteome that fully explains a cryo-EM map. This holds true even for novel protein folds for which no prior structural information exists. Prior to 2020, this identification process was challenging without deep-learning-based tools (see below), but possible. One approach involved narrowing down protein candidates by comparing secondary structure predictions of all proteins to manually traced models [57], followed by full-length threading of selected sequences into the map to determine compatibility with the cryo-EM map [58]. Another strategy involved matching side chain lengths from the cryo-EM map to a library of mass spectrometry-detected proteins [59].

Since 2020, new deep-learning tools have greatly simplified this protein identification step. Advancements in protein structure prediction, particularly with AlphaFold [60], enabled the comparison and matching of traced backbones against libraries of AlphaFold predictions [61]. Additionally, deep-learning methods like DeepTracer [62] and ModelAngelo [63] began to automate backbone tracing from cryo-EM maps and allow programs to ‘guess’ residues based on side chain density and other factors, often surpassing expectations in their high accuracy. For instance, DeepTracer currently can guess residues to produce sequences with higher than 40% identity with the correct protein sequence from a ~3.5 Å sharpened map. This has made it possible to directly BLAST the guessed amino acid sequence against a genome, or even the whole UniProtKB database, to identify the protein. ModelAngelo demonstrates even higher accuracy than DeepTracer, not only in terms of the correctness of guessed residues (often higher than 50% identity for a ~3.5 Å map) but also by listing the likelihood of all residues to enable a more powerful BLAST search [63]. Interestingly, both DeepTracer and ModelAngelo seem to achieve higher amino acid prediction accuracy when the cryo-EM map is sharpened by the traditional B-factor method, rather than by recently developed deep learning-trained methods. The B-factor [64], adapted from the temperature factor in X-ray crystallography, measures how much a cryo-EM map loses its signal as resolution increases. Sharpening the map involves a mathematical adjustment: it uses the B-factor to boost the high-frequency data that correspond to the fine map details.

With these advancements, cryo-EM emerged as an ‘open-minded’ approach for studying microbial appendages, without knowing the identity of proteins in a mixed sample. Multiple appendage types can be mechanically sheared from cells and imaged together without additional purification. For example, using samples sheared from electrode-grown *G. sulfurreducens* biofilms, cryo-EM identified multiple filaments and solved the first structure of the hexaheme OmcS nanowire from this mixture [26] by manually threading possible sequences into the cryo-EM map. Even though *G. sulfurreducens* encodes three other hexaheme cytochrome homologs closely related to OmcS, the cryo-EM map contained enough side chain densities to identify which protein comprised this filament amid the other contaminants.

Cryo-EM was then able to solve the structures of the remaining unidentified appendages present on *Geobacter* cell surfaces. A *ΔomcS* strain allowed identification of a thinner tetraheme OmcE cytochrome filament [24], and a ∆*omcSE* mutant led to an OmcZ nanowire structure [25]. Both the OmcS and OmcZ structures have been independently verified in other strain backgrounds [27,28]. A hypothetical OmcZ model had been deduced earlier from nanoscale IR (nano-IR) spectroscopy [65], but this model is inconsistent with the experimentally determined cryo-EM structure (RMSD of 28 Å over 243 atoms). This is consistent with the typical applications of nano-IR spectroscopy, which is primarily used to probe protein secondary structure rather than to obtain 3D structural models. Other than different protein nanowires, an elusive 3 nm filament present in many sheared preparations, proposed to be the PilA-N ‘e-pilus,’ was determined to be B-DNA by two different groups [29,66]. Structures for larger diameter (7–8 nm) filaments were shown to be T4P composed of both PilA-N and PilA-C [24,29]. To date, no other appendage structures have been observed using high-resolution cryo-EM in *G. sulfurreducens*, but it is plausible that more appendages will be discovered as more growth conditions and strains are investigated.

**Figure 1:**
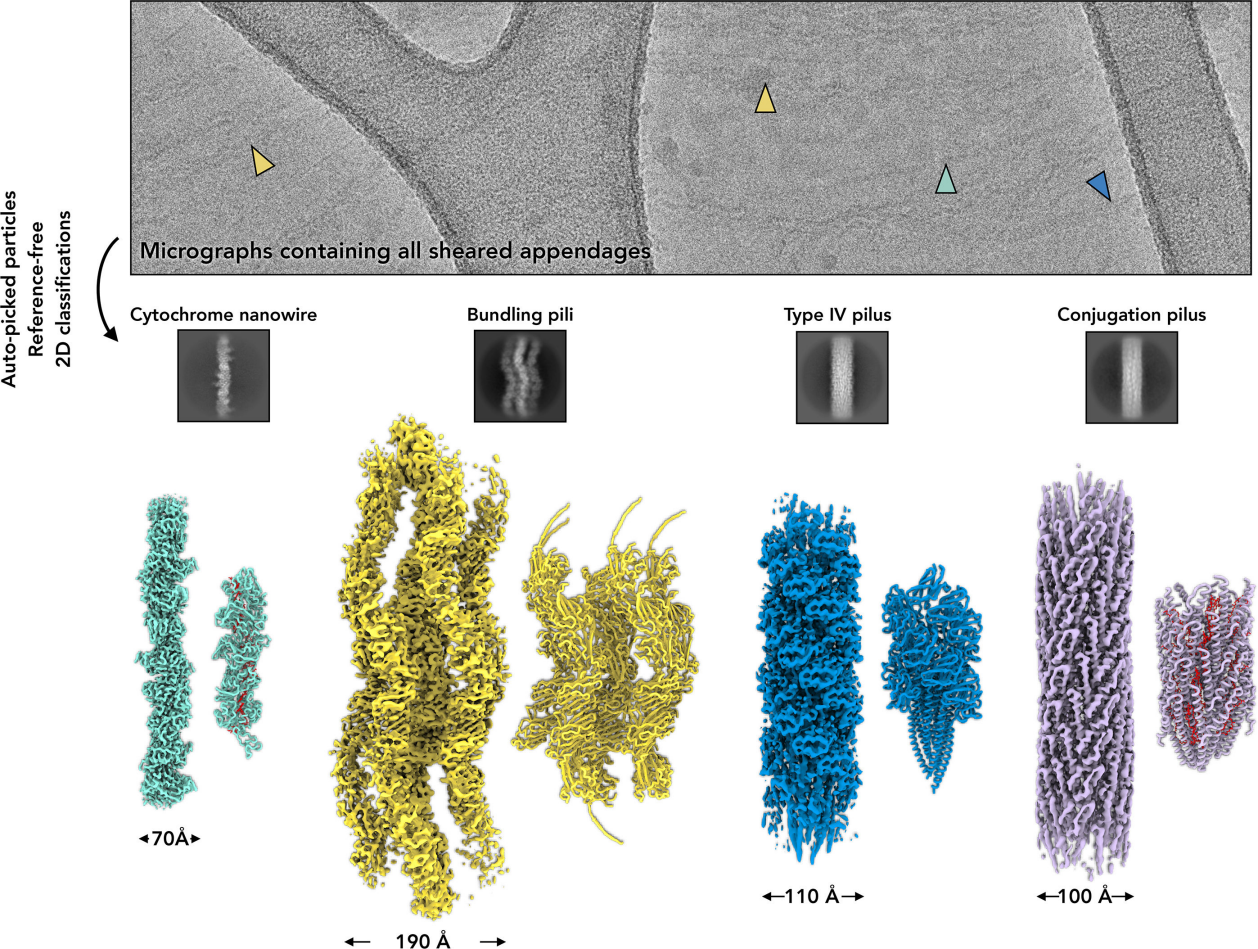
Cryo-EM of diverse extracellular appendages from *P. calidifontis.* A representative cryo-EM micrograph of sheared appendages from *P. calidifontis* reveals a variety of extracellular appendages (top). These appendages were mixed together after mechanical shearing and imaged on a lacey carbon grid. Different appendage types are visible, including cytochrome nanowires [67] (cyan, PDB 8E5F), archaeal bundling pili [68] (yellow, PDB 7UEG), archaeal type IV pili [69] (blue, PDB 8FK7), and conjugation pili [70] (lavender, PDB 8DFT). To analyze these diverse structures, all filament segments were automatically picked from approximately 10,000 such micrographs. Reference-free 2D classification was employed to sort the extracted particles, a process that can be iteratively performed. Those resulting classes were then visually inspected, and those corresponding to the same pilus type were grouped for subsequent analysis. Each individual subset was then used for helical symmetry testing and 3D reconstructions (bottom).

The approach developed for *Geobacter* has also begun to discover unique cytochrome appendages in Archaea. For instance, four different appendages were observed in samples sheared from *Pyrobaculum calidifontis* cells and subsequently imaged by cryo-EM without purification. Those four types of appendages were sorted by reference-free 2D classification, a process where extracted particles are grouped into classes based on their visual similarity without using any external references. This step allows users to sort different pili into subsets based on their appearance, such as diameter, lumen size, or repeating patterns observed in real space and in the averaged power spectrum. After grouping the different pili into subsets, each one can then be reconstructed separately to near-atomic resolution. At this resolution, protein backbones and even most side chains can be distinguished, which allows their identities to be conclusively determined (Figure 1) by the methods discussed above. The four different appendages included a new family of cytochrome filament with a similar densely packed heme core [67], a conjugation pilus [70], flagella-like type IV pilus [69], and an archaeal bundling pilus [68]. A similar effort using *Archaeoglobus veneficus* produced another unique cytochrome filament [67].

### The role of heme arrangement in long-range electron transport

CryoEM has played a pivotal role in advancing our understanding of the significance of heme arrangements in electron transfer by anaerobically respiring microbes. The dense arrangement of heme within multiheme cytochromes was suggested over two decades ago to be a key feature directing the flow of electrons, based on protein crystal structures [71,72]. As more multiheme cytochrome crystal structures were determined, it was noted that many of those involved in microbial respiration shared a conserved arrangement and heme pair geometry [73–75]. The crystal structure for periplasmic flavocytochrome *c* fumarate reductase, the first protein structure deposited in the PDB from a metal-reducing bacterium, highlighted the role of such heme chains in shuttling electrons as part of anaerobic respiration in *S. oneidensis* [76]*.* Soon thereafter, the newly determined structure of its periplasmic small tetraheme cytochrome (STC) was used to suggest that chains of similarly co-ordinated heme *c* ligands were acting as ‘electron harvesting’ networks allowing these proteins to act as shuttles of bidirectional electron transfer between non-specific donors and acceptors [77]. The earliest *G. sulfurreducens* protein in the PDB, the triheme PpcA, is also a periplasmic electron shuttle critical for respiration of acceptors outside of the cell [78]. This structure also pointed to a model in which the dense arrangements of heme within a cytochrome determined its ability to transport electrons through, and between, cytochromes. In particular, these structures all exhibited hemes that formed contiguous chains in pairs of alternating parallel and perpendicular arrangements [79].

Up to this point, however, little was known about the structures responsible for the transport of electrons from organisms to extracellular electron acceptors on the cell surface. The first experimentally determined structure of these extracellular electron transfer proteins was the crystal structures of *S. oneidensis* terminal reductase MtrF [80], and this was later followed by the X-ray structure of the entire transmembrane, three-protein complex of MtrABC [81]. The electron-transporting components of these proteins were also heme chains, and it was noted that the ten-heme arrangement of the transmembrane ‘wire’ (MtrA) mirrored that of previously established structures of *S. oneidensis* STC and *E. coli* NrfB, a mediator cytochrome for electron shuttling to the periplasmic cytochrome *c* nitrate reductase NrfA. The determination of the MtrABC complex structure, along with biochemical data, conclusively established the protein acted as a conduit using a chain of heme cofactors from the periplasm directly to the exterior of the cell. The enormous size of the above-mentioned *G. sulfurreducens* conductive appendages, first imaged [18] at low resolution by TEM in 2005, precluded crystallization, and their structures would remain unknown until cryo-EM methods were brought to bear on the question of their protein identity 14 years later [24–28], as detailed in the preceding section.

Beyond identification of the extracellular appendages of *G. sulfurreducens* as cytochrome wires, cryo-EM structures inform our understanding of the role of heme arrangements in supporting long-range electron transport. The dense packing of heme in *G. sulfurreducens* cytochrome wires follows a motif where their porphyrin planes are in parallel (staggered co-facial) or obtuse (‘T-shaped’) angle pairs. This same motif is found in non-wire-forming multiheme cytochromes, such as those discussed above. Homologs of OmcS, OmcZ, and the archaeal A3MW92 and FK2MU8 (from *P. calidifontis* and *A. veneficus*, respectively) wire-forming cytochromes are found in diverse species within bacterial and archaeal domains of life [67], and similar heme arrangements are conserved in a diversity of other non-filamentous cytochrome structures [82]. A meta-analysis of all heme pairs in all multiheme c-type cytochrome structures deposited in the Protein Data Bank shows that the vast majority of these pairs are packed in such parallel or T-shaped arrangements [67]. The broadly conserved geometry of these heme pairs suggests a convergent design rule evolved to control electron transfer through multiheme c-type cytochromes.

Despite the seemingly simple geometrical constraints elucidated by cryo-EM and X-ray crystallography of multiheme c-type cytochromes, our understanding of their role in determining electron transfer and long-range electron transport remains lacking. For instance, calculations of the expected electronic conductivity of OmcS using a conventional, semi-classical Marcus theory approach fall orders of magnitude short of experimental observations [83,84]. Chemical intuition suggests that electronic coupling is greater between parallel stacked heme pairs as compared with T-shaped pairs due to greater orbital overlap. This conclusion is somewhat borne out in computational approaches estimating electron transfer rates in OmcS structures, where T-shaped pairs are predicted to be the rate-limiting step in electron transport through OmcS oligomers [85,86]. However, computed coupling energies between both types of heme pairs in multiheme cytochromes in general are small [87], and the thioether bonds between heme *c* and the protein Cys residues contribute to greater-than-expected electron transfer rates between T-shaped pairs [88,89]. Despite the small predicted electronic coupling values, actual electron transfer rates between some heme pairs in *S. oneidensis* MtrC were measured experimentally to be among the highest values observed for any multiheme cytochrome, and with, counterintuitively, similar rates between parallel and T-shaped heme pairs [90]. A recent meta-analysis of published spectroscopic data along with new electronic structure calculations suggests that different electron orbitals may be involved in electron transport between electrodes as compared with those involved in solution redox kinetics, concluding that some greater electronic coupling may be present in the former case [91]. Newer quantum mechanical models for electron transfer in cytochrome nanowire conduction [92–95] also suggest that the mechanisms of charge transport may not be the pure hopping model implicit in Marcus theory. Nevertheless, more efforts are required to fully reconcile experimental and theoretical observations of electron transport in cytochromes to improve our understanding of the structure-property relationships of these conserved heme arrangements in sustaining conductivity through proteins.

In addition to the spatial arrangement of heme, the co-ordination environment, elucidated by cryo-EM and other structural methods, can affect their electron transport properties. For example, another common feature of cytochrome wires, and of the other multiheme cytochromes discussed above, is that all hemes in these proteins are bis-His co-ordinated. The porphyrin and two axial co-ordination bonds to the His imidazole group represent the primary co-ordination sphere of the heme Fe. Since these are nominally identical in all heme in these cytochromes, any differences in redox potential must be due to the surrounding environment of either the protein or the solvent, termed the secondary co-ordination sphere [87,96]. Cryo-EM models of OmcS from cryo-EM allow molecular dynamics simulations of the heme primary and secondary co-ordination spheres. Such simulations are used to determine energy-minimized or mean molecular environments of the heme from which to calculate redox potentials and coupling energies of the heme chains [85,86]. The general conclusion of these calculations is that the potential energy surface over which electrons traverse extended heme chains is surprisingly corrugated. Some heme–heme redox potentials differ by more than 200 mV, a value much greater than thermal free energy available at room temperature. This suggests protein conformation and structural dynamics may also influence electron transfer rates. Conformational fluctuations can alter both His imidazole orientation relative to the porphyrin ring and the heme orientation, both of which can affect electron transfer [97]. Protein conformational changes can also affect the planarity of the heme porphyrin ring, with ruffled hemes stabilizing the ferric (Fe^3+^) state and potentially impacting electron transfer [86,98].

**Figure 2:**
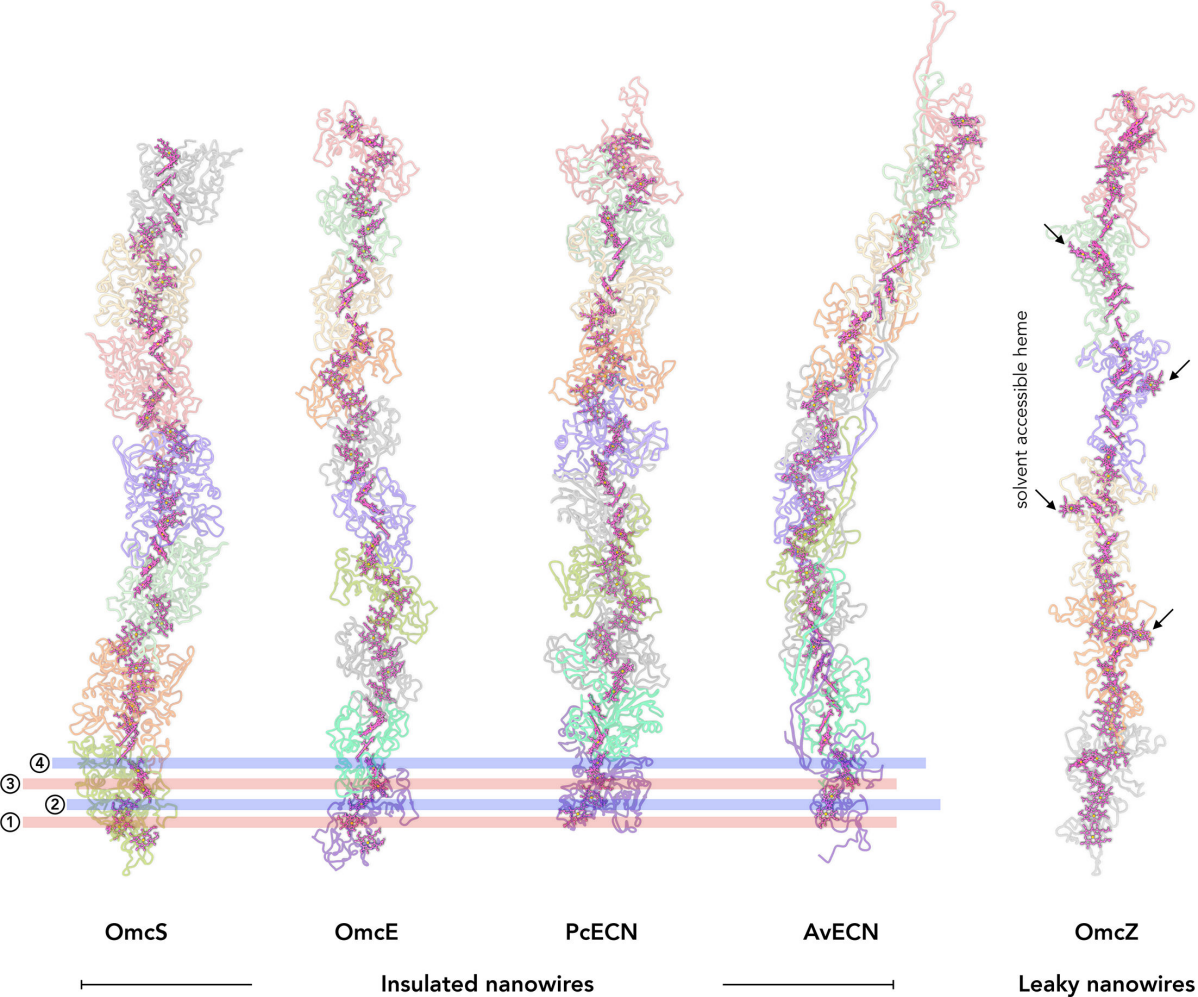
Heme arrangements in known cytochrome nanowires. This figure displays five distinct cytochrome nanowires: OmcS [26] (PDB 6EF8), OmcE [24] (PDB 7TFS), PcECN [67] (PDB 8E5F), AvECN [67] (PDB 8E5G), and OmcZ [25] (PDB 8D9M). The first four are classified as ‘insulated’ nanowires, while OmcZ is considered ‘leaky.’ Each protein subunit within a nanowire is represented in a different color, with the polypeptide backbone shown with transparency. Heme molecules are displayed in pink using a ball-stick representation. A conserved arrangement of four hemes (stacked → T-shaped → stacked) is highlighted within the insulated nanowires, indicated by transparent blue and red stripes. In contrast, the solvent-accessible hemes in OmcZ are indicated to illustrate their distinct arrangement.

Lastly, the cryo-EM structure of cytochrome wires provides new clues about their evolution and physiological roles. For example, the *G. sulfurreducens* OmcS and OmcE wires, despite a lack of sequence and structural homology and comprising a hexa- and tetra-heme cytochrome subunit, respectively, both have an interfacial heme co-ordinated by one His in one subunit and another His in the adjacent subunit. OmcZ, on the other hand, has all eight hemes in the wire subunit co-ordinated by His within the same subunit, suggesting a distinct evolutionary trajectory [25]. Based on the solvent accessibility of their hemes, *G. sulfurreducens* filaments and the two known archaeal cytochrome structures can be further classified into two subgroups: ‘insulated nanowires,’ where hemes are shielded from solvent by the protein component (e.g. OmcS, OmcE, PcECN, and AvECN); and ‘leaky nanowires’ (e.g. OmcZ), where at least one heme per subunit is solvent-exposed (Figure 2). For OmcZ, wires of which are produced primarily when *G. sulfurreducens* is grown with a solid anode poised at an appropriate oxidizing potential, this leaky structure could potentially contribute to the formation of conductive networks of wires within biofilms by minimizing the physical barrier for electron hopping between OmcZ filaments within a mesh [25]. For the insulated wires, on the other hand, how their reactivity towards environmental oxidants differs from OmcZ and each other remains an open question. Cytochromes, and especially secreted cytochrome wires, are biosynthetically expensive biomolecules, so it stands to reason that each has a somewhat distinct role in the physiology of the organisms that produce them. However, these organisms did not evolve in electrochemical cells, so determining the role of OmcZ in the environment, let alone the roles of OmcS, OmcE, PcECN, and AvECN in the anaerobic respiration of these organisms, is an outstanding challenge.

### Linking new cytochrome structures to published literature

An unfortunate fact arising from the discovery that multiple cytochrome nanowires, pilin structures, and extracellular DNA could be co-present in sheared preparations is that much early data are based on cells expressing multiple filaments or producing mixtures of unidentified proteins. In many cases, ‘wild type’ strains used by different labs overexpress some of these filaments, and laboratory evolution tends to select for loss of these expensive structures. Debates about this literature and the roles of various nanowires in prior studies still persist [66,99,100]. Is it possible to re-examine electron micrographs in published literature, such as negative staining images with high image dots per inch (DPI), to identify the specific filaments shown? In many cases, this is indeed possible, especially due to the distinctive sinusoidal morphology of known cytochrome nanowires.

**Figure 3:**
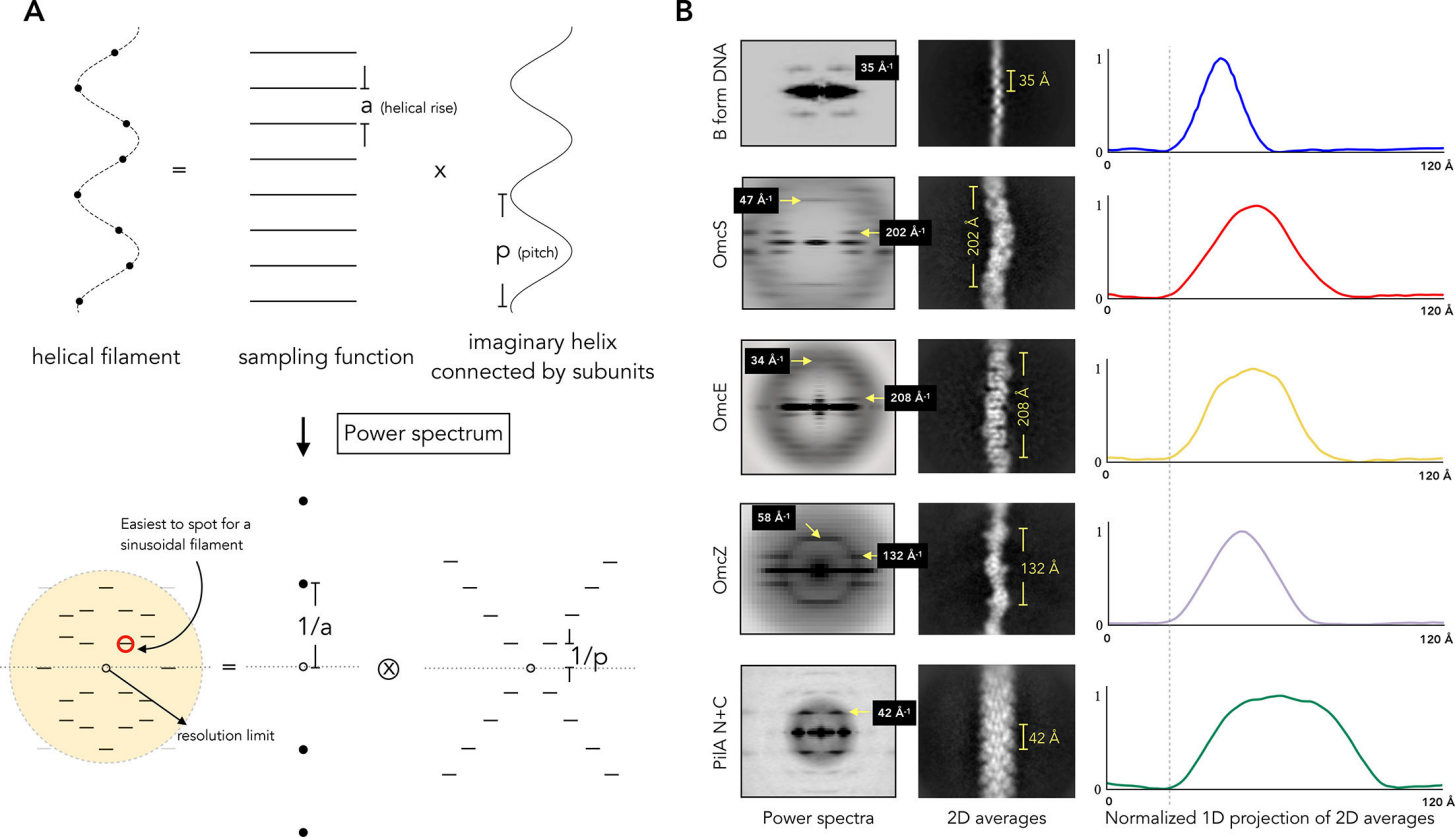
Low-resolution helical features of extracellular filaments from *G. sulfurreducens.* (**A**) All known cytochrome nanowires and B-DNA can be roughly viewed as simple filaments formed of a single protofilament (top). Cryo-EM produces a 2D projection of a 3D object. Thus, a helical filament (in 2D projection) under a cryo-EM micrograph can be considered the multiplying product of two functions: a sampling function spaced apart by the helical rise and a cosine wave with a period corresponding to the helical pitch. The power spectrum of the sampling function is a row of spots along the meridian. The power spectrum of the cosine wave is a series of layer lines, spaced 1/pitch apart, which form a cross centered on the origin. The power spectrum of the helical structure is the convolution of the transforms of the continuous helix and the sampling function. Information about helical pitch and rise can be found at the same spot from those two individual power spectra before convolution. When processing experimental data, visible layer lines are affected by the resolution limitation. (**B**) The power spectra (left) of B-DNA [24], OmcS [26] (PDB 6EF8), OmcE [24] (PDB 7TFS), OmcZ [25] (PDB 8D9M), and type IV pilus [26] (PilA-N + PilA-C, PDB 7TGG) are shown. These are averaged power spectra generated from aligned raw particles imaged by cryo-EM. Their corresponding 2D averages are shown in the middle. Projecting those 2D averages onto the bottom line and normalizing their intensities to (0, 1) yields the one-dimensional (1D) signature plot of those filaments. These peaks were aligned at the left starting point.

Consider a simple helical structure formed of a single protofilament (Figure 3A), in which a single strand of proteins forms a helical filament, representing all currently known cytochrome nanowires. Therefore, we can view these one-protofilament helical cytochrome nanowires as the product of two functions: (a) a sampling function with intervals corresponding to the helical rise and (b) a continuous helix formed by connecting helical subunits [101]. These functions are typically analyzed using a power spectrum, which visualizes the periodic information in Fourier space. For example, the power spectrum of function (a) reveals the helical rise in reciprocal angstroms along the meridian. The power spectrum of function (b) provides the helical pitch information, derived from the filament’s sinusoidal shape. The power spectrum of the helical structure is the convolution of these two power spectra. Consequently, if the DPI in the original publication is sufficiently high, both the helical rise and pitch of the imaged filaments can be estimated using this method.

The helical rises of OmcS, OmcE, and OmcZ are 47 Å, 34 Å, and 58 Å, respectively. These features might be difficult to spot in published micrographs taken under a low magnification. However, the helical *pitches* of OmcS, OmcE, and OmcZ are larger (202 Å, 208 Å, and 132 Å, respectively). Thus, if a sinusoidal shape can be observed and the figure has an accurate scale bar, the pitch of those filaments can be measured and compared with known filamentous structures. One way to accomplish this is to first save the literature figure in an image format supported by EMAN2 [102]. First, individual filaments are selected from the main image using a specialized tool (e.g. e2helixboxer). This program digitally cuts out each selected filament and rotates it to stand perfectly vertical, saving each one as a separate image. Next, each of these vertical filament images is placed into the center of a larger, blank square—a technique known as ‘padding.’ This step is essential preparation for generating a ‘power spectrum,’ which is a visual map that reveals the filament’s repeating patterns. By adding spectra together to improve the signal, we have re-analyzed micrographs reported to contain ‘e-pili’ [103] and demonstrated that these filaments share the same helical symmetry as OmcS cytochrome nanowires [26].

For filaments with a short pitch, such as B-DNA and type IV pilus PilA *N* + C (Figure 3B), layer lines may not be visible in a single negative staining micrograph due to limited resolution. However, further analysis is possible if raw cryo-EM micrographs from previously published studies are available in the Electron Microscopy Public Image Archive (EMPIAR). Specifically, examining the average power spectrum, 2D averages, and one-dimensional (1D) projections of those averages can yield valuable insights. As discussed earlier, the average power spectrum reveals repeating features of the filament. In cases where a large number of high-quality particles are available, generating high-resolution 2D averages can reveal the unique feature of the fiber, which can then be compared with other known structures.

However, for flexible filaments like B-DNA, where limited particle numbers may hinder the effectiveness of power spectra and 2D averaging, we found it is insightful to examine the 1D projection of the 2D average (Figure 3B). This approach can be particularly useful because it provides a filament signature even when resolution is limited. We used this method to examine the 19 fibers present in EMPIAR-11228 proposed to be ‘e-pili’ and conclude they are consistent with B-DNA filaments [66]. It’s important to note, however, that this method doesn't measure the filament diameter. In the 1D projection, each point on the line represents the sum of intensities in the 2D average along the helical axis at that position. This means that the measured value will be larger than the actual diameter if the filament has a sinusoidal morphology, as is the case with B-DNA and cytochrome nanowires.

In conclusion, rapid advancements in cryo‐EM are expanding our understanding of how microorganisms conduct extracellular electron transfer by revealing new families of cytochrome-based filaments. These structures allow reanalysis of historical electron micrographs to clarify some past ambiguities and add information to prior studies. The heme cores of cytochrome nanowires suggest that convergent solutions exist for efficient long-range conduction and reveal both conserved heme pair geometries and variations in solvent exposure. Discrepancies between computed electronic coupling and observed electron transfer rates suggest that secondary co-ordination effects or protein dynamics remain to be resolved. Further research will also be needed that combines high-resolution structural, computational, and *in vivo* approaches with careful mutant construction to elucidate the physiological roles of cytochrome nanowires in microbial electron transport.

**Summary points**

- *Geobacter sulfurreducens* reduces insoluble electron acceptors using microbial nanowires in a process known as extracellular electron transfer.
- The resolution revolution in cryo-EM and deep-learning modeling tools allows the building blocks of microbial nanowires in *G. sulfurreducens* to be identified *de novo* as multi-heme c-type cytochromes.
- Despite the lack of homology among known cytochrome nanowires, all of the cytochrome nanowires share a similar heme arrangement for electron transfer.
- Caution is needed when interpreting previously published experimental data, given the possibility of filament misidentification and differences in strain backgrounds.

## Open Access

This article has been published open access under our Subscribe to Open programme, made possible through the support of our subscribing institutions, learn more here: https://portlandpress.com/pages/open_access_options_and_prices#conditional

## Abbreviations

DPI: dots per inch
EMPIAR: Electron Microscopy Public Image Archive
SHE: Standard Hydrogen Electrode
STC: small tetraheme cytochrome
TEM: Transmission Electron Microscopy
T4P: type IV pili
nano-IR: nanoscale IR
